# Shaping the Future of Men’s Health: How AI Could Be a Transformative Tool for Better Patient Outcomes and Provider Efficiency

**DOI:** 10.2196/81314

**Published:** 2026-07-23

**Authors:** Bryce Bisset, Vasanth Kainkaryam, Shawn Marhamati, Amin Moradi, Reza Amin, Justin Houman

**Affiliations:** 1 Bastion Health, 400 Farmington Ave, Farmington, CT, United States, 1 650-610-1616; 2 Urology Department, Potomac Urology, Alexandria, VA, United States; 3 Urology Department, Cedars-Sinai Medical Center, Los Angeles, CA, United States; 4 Urology Department, Tower Urology, Los Angeles, CA, United States

**Keywords:** artificial intelligence, artificial intelligence agent, agentic AI, urolgoy, men's health, men's urology, healthcare AI, telemedicine, quintuple aim, healthcare equity, provider experience, patient experience, public health outcomes, healthcare costs

## Abstract

Men’s health urology faces growing challenges driven by workforce shortages, rising disease burden, and persistent disparities in care. Despite an increasing prevalence of conditions like benign prostatic hyperplasia, prostate cancer, and urinary tract infections, more than half of U.S. counties lack a practicing urologist. Stigma, access, and uncertainty lead men to delay care further, resulting in higher morbidity, late-stage diagnoses, and unsustainable costs. To meet these challenges, this paper explores the transformative potential of agentic AI systems to drive Healthcare 5.0 in urology to create a more equitable, efficient, and proactive care system. We examine how AI can advance the quintuple aim of health care: enhancing patient experience, improving population health, reducing costs, increasing provider satisfaction, and promoting health equity. The paper introduces the concept of a suite of specialized AI agents, rooted in both currently in use and developing AI applications, that work collaboratively to support providers, patients, and health care administrators across the continuum of care. These agents not only improve efficiency, streamline workflows, and augment clinical reasoning, but also enable scalable, virtual-first care delivery systems. We articulate our view of the future urology patient journey, illustrating how AI agents can transform each step of the process to provide an improved, seamless experience for patients and providers while maintaining human-centered, personalized care. Finally, we outline critical future directions, such as data interoperability, regulatory frameworks, and inclusive design principles, to ensure that AI technologies are deployed safely, equitably, and in line with ethical regulations. Through the strategic implementation of agentic AI, we view the future of men’s health urology as a model for innovation, driving better outcomes for patients and sustainable, meaningful care for providers.

## The Challenge in Men’s Health Urology

### Overview

Urology plays a central role in addressing prevalent and consequential health conditions among men. Benign prostatic hyperplasia, urinary tract infections, urolithiasis, bladder cancer, kidney cancer, and prostate cancer are 6 of the most prevalent and costly urologic conditions that impact patient quality of life and life expectancy [1,2]. Despite this importance, significant disparities in access persist: over 62% of US counties lack a practicing urologist, and more than 50% of men delay or avoid seeking care due to stigma, logistical barriers, or lack of awareness [3,4].

Delays in diagnosis and treatment are a well-documented driver of excess morbidity, mortality, and health care expenditure [5,6]. Dependence on limited human resources is impacted by a growing shortage of urologists, leading to suboptimal scheduling practices, such as lower-acuity conditions or stable follow-up patients consuming valuable clinic time. As a result, new patient consultations for potentially high-risk or time-sensitive urologic conditions are subjected to prolonged wait times. This could lead to later recognition of diseases, such as late-stage prostate cancer management, which is significantly more costly and less effective than early intervention [5,7]. These delays represent not only a clinical failure but also a major inefficiency within the broader health care system.

Furthermore, the incidence of conditions like benign prostatic hyperplasia and urinary tract infections has increased dramatically over the past few decades [8]. Prostate cancer is also the second most common cancer and the fifth leading cause of cancer mortality in men [9]. Bladder and kidney cancers respectively rank the 9th and 14th most dangerous cancers worldwide [9]. To address the high prevalence of these conditions, the importance of access to expert physicians to discuss effective screening techniques that result in improved patient outcomes is essential.

Traditional care models built on outdated technology, a dependence on in-person visits, and limited accessibility are unsustainable within a changing health care landscape. To address this, a more scalable, proactive, and intelligent model is required. AI, particularly in the form of agentic clinical systems, demonstrates the potential to drive systemic change in health care to reach Healthcare 5.0 and fulfill the goals of the quintuple aim.

The aim of this paper is to review the potential of early-stage agentic AI tools to innovate health care systems, specifically in urology settings. We highlight examples of how these tools could be used to reach Healthcare 5.0 in urologic settings and fulfill the goals of the quintuple aim. Specifically, we focus on the current burdens on the system, the importance of technological innovation, and the potential for AI agents to drive this innovation. This viewpoint is tailored for physicians, urologists, clinical administrators, and health care technologists focused on scalable solutions within the men’s health space.

### AI as a Catalyst for the Quintuple Aim

The quintuple aim of health care describes the framework guiding necessary advancements within the health care system. It points to patient experience, population health, costs, provider experience, and health equity as the 5 domains of the health care system that must be improved [10]. AI tools offer a promising solution for advancing medical goals, enabling greater accessibility and scalability to help systems adapt to the growing demands of an evolving landscape [11].

#### Patient Experience

Improved patient experience is shown to dramatically improve patient health outcomes and health insurance systems [12,13]. AI-driven systems can curate a personalized experience, deliver efficient triage and intake, and provide educational interventions that encourage early engagement, improve accessibility, and close gaps in care [14,15]. By using an agentic AI approach, it further removes any stigma that men may feel by trying to identify their specific needs and catering to their approach [16,17]. Examples include customizing an intake form as a conversation, providing real-time educational information, suggesting questions that the patient may ask during their visit based on information they have provided, providing validation and encouraging conversation, and others.

#### Population Health

AI tools have demonstrated the capacity to positively impact population health [18,19]. By improving efficiency, identifying at-risk populations, enhancing research, and streamlining workflows, AI can contribute to better safety, quality, and consistency of care in health care delivery [20]. Rather than relying on reports, AI can assist in proactively working with the physician in order to address population health, including prompts, suggestions, preparation of orders, and more [21,22]. AI can also evaluate individual demographic data, reviewing it against clinical studies to identify whether population health data will apply to a specific individual and their medical context [23,24].

#### Cost Containment

$760 to $900 billion USD is considered to be due to waste in the US health care system [25]. Predictive and preventative care systems can minimize avoidable acute care, address costly chronic diseases early, and reduce redundant testing [26,27]. AI-driven systems can assist in identifying disease and optimized care navigation to ensure accurate and necessary follow-up, preventing excessive services [28,29]. Furthermore, AI tools can assist in administrative tasks, improving provider efficiency and ensuring consistently accurate billing and coding [30,31]. Examples include ensuring laboratory results are available at the time of office visits, following through on referrals, querying databases to ensure clinical data is synthesized for ease of physician use, and others, contributing to a potential US $200-$360 billion in savings on health care spending [32].

#### Health Equity

AI tools can improve health equity by increasing access, breaking down language barriers, and supporting targeted interventions [33,34]. Through large-scale data analysis, AI tools can detect inequities in resource allocation and direct health care strategies towards marginalized populations [34-36]. Virtual, mobile, and telehealth platforms offer a scalable mechanism to provide access to patients typically excluded from traditional care delivery systems [37,38].

#### Provider Experience

AI-driven systems reduce administrative load, streamline navigation, and facilitate more timely and personalized care delivery [15,38]. While patients benefit from timely and personalized care delivery that improves engagement and outcomes, providers get to experience a much more meaningful interaction with their patients by removing clerical and administrative tasks so that their focus is on the clinical interaction and rapport building, augmented by AI prompts and suggestions from clinical studies [30]. In this way, AI tools can improve provider efficiency, improve diagnostic accuracy, and reduce the burden of administrative tasks and documentation to decrease rates of burnout [15,39].

### The Role of AI in Driving Healthcare 5.0

**Figure 1.**
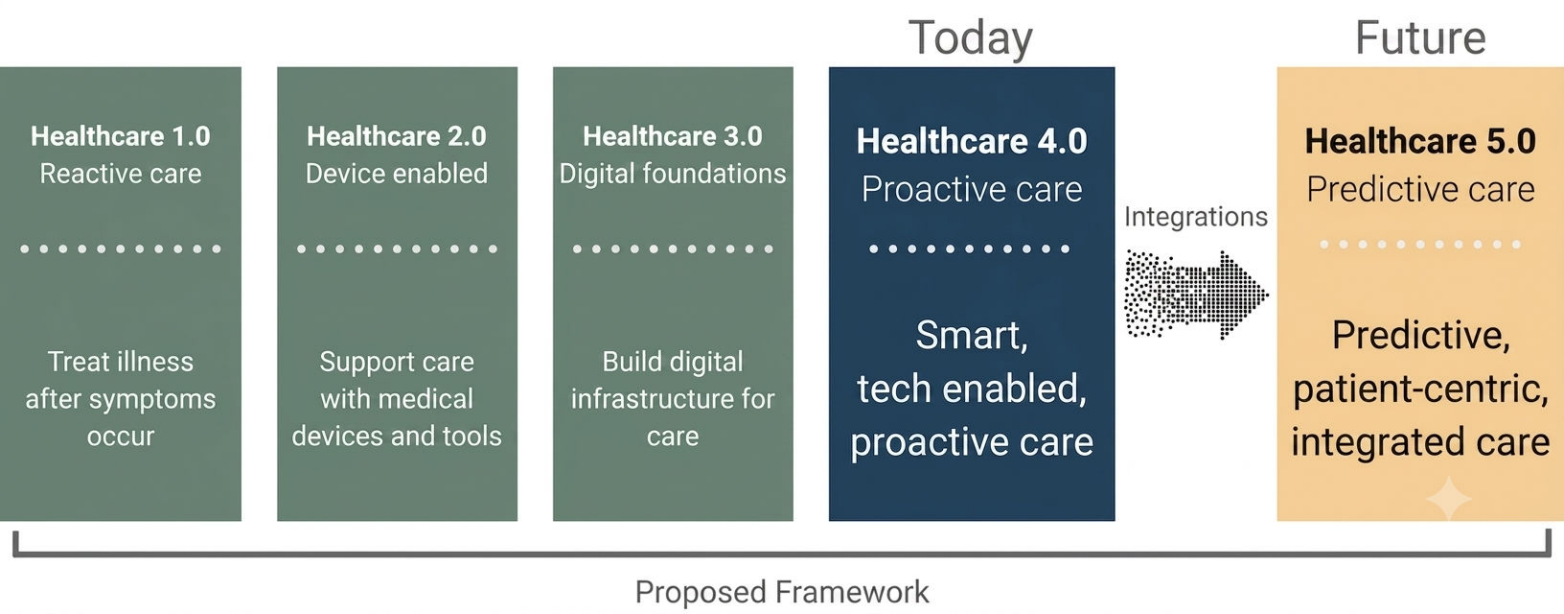
A proposed workflow to describe previous, current, and future stages of health care systems. Each stage is identifiable by the technology available at the time and the approach to patient care [15,40-44].

Healthcare 5.0 describes the next generation of health care systems defined by a digital transformation beyond Healthcare 4.0. Where Healthcare 4.0 was marked by innovations such as telehealth, remote patient monitoring devices, and early disease detection and prevention, Healthcare 5.0 introduces AI and machine learning, the Internet of Things, and smart devices that enable highly personalized patient-centric care [40,41]. Achieving Healthcare 5.0 requires a transition that embraces technological advancements, recognizes the value of predictive care, and improves health care systems. The transition from previous systems to Healthcare 5.0 is depicted in Figure 1.

Telehealth, integrated care, and the use of AI are elements necessary to meet the demands of a changing health care landscape, bridging the gap between today’s health care and the future [42]. AI agents are tools well equipped to enhance health care delivery systems, supporting patients, providers, and administrators in addressing the goals of the quintuple aim [15,41].

## Introducing the Agentic Model in Health Care

### Overview

**Figure 2.**
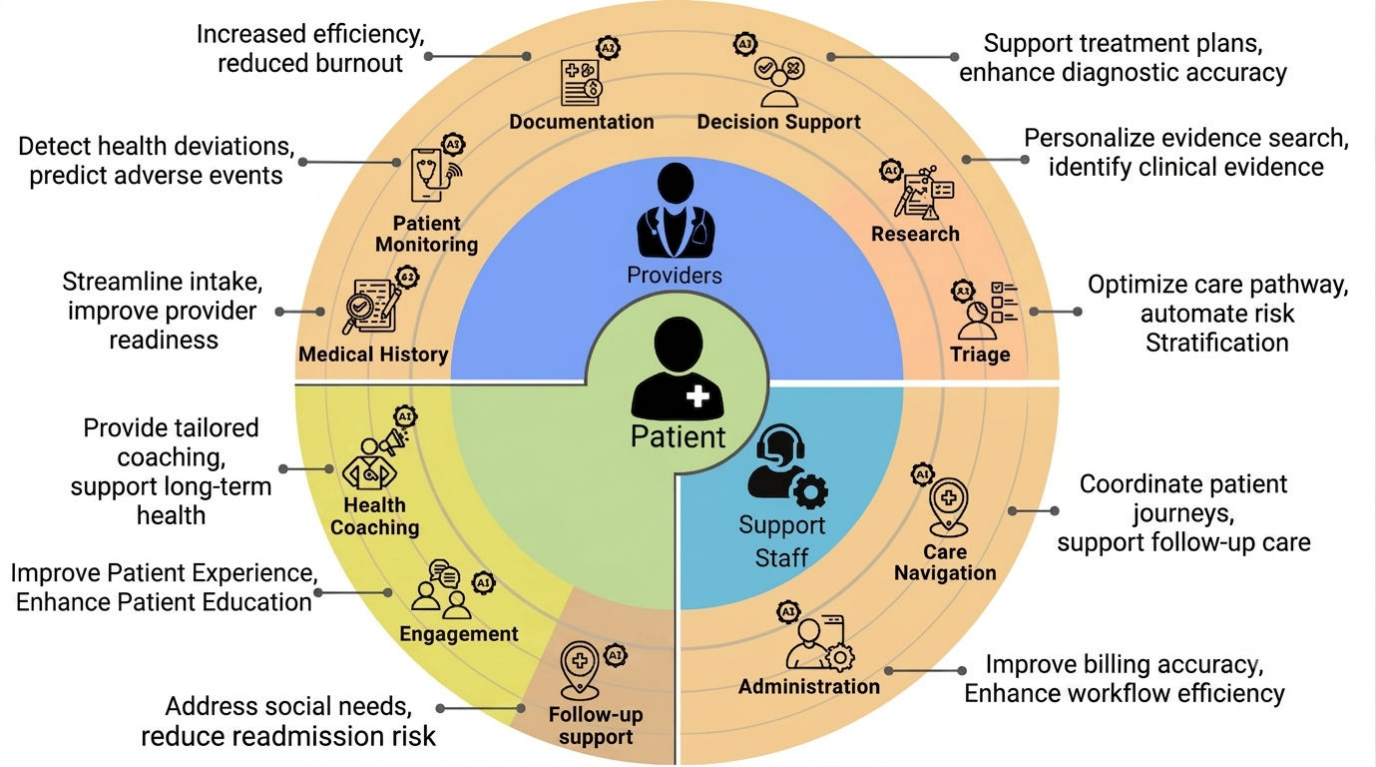
A proposed schematic to depict AI agents supporting patients directly and assisting providers and support staff in delivering patient care [22,43,46-48].

Agentic AI models are promising innovations that incorporate the robust capabilities of traditional large language models (LLMs) with specialized tools that enable task-specific problem solving. These individual AI agents work cooperatively across a distributed network to allow for a continuum of care across health care systems [45]. Figure 2 demonstrates the interactions between humans, between AI, and between AI and humans in an integrated technological ecosystem.

Specialized analytical tools and the capability to conduct research, integrate external knowledge, and access long and short-term memory intelligently allow task-specific roles to be filled within health care systems. In urology, many such roles could be filled by AI agents.

### AI Agents

AI agents are role-specific support tools that can operate in specific capacities, allowing for greater functionality and task-efficiency than traditional LLMs. While high-quality, prospective longitudinal studies comparing AI-assisted health care systems and traditional care delivery systems remain in their infancy, there is mounting evidence that the presence of AI tools in health care could increase over time [49,50]. We describe AI agents that are specialized to fit specific roles within these future health care systems.

#### Engagement Agents

Engagement agents interact with patients through personalized communication with the goal of improving patient adherence to care plans, enhancing patient education, and providing reminders [51,52]. Current chatbots can tailor their communication to the preference and style of patients to keep conversations relevant and engaging for the patient [52]. In addition, these agents could cross language barriers by providing real-time translation between languages and adjusting the information presented to educational and reading levels [53]. They can facilitate patient participation in their health, encouraging patients to take preventative measures and easing the burden on providers [51]. Engagement agents utilizing these tools demonstrate the ability to have feasible and positive effects on patient outcomes [14,54].

One example of such a feature is Woebot, a LLM-based chatbot for patient engagement that analyzes emotional states in the conversation and makes adjustments to deliver context-aware responses in real time [51]. Technologies such as these demonstrate the ability to maintain quality and empathetic care delivery while easing the burden on providers [14,55]. A practical example in a urologic setting would be a patient portal, in which a patient can exchange secure messages with an engagement agent. As seen in a Stamford University Study, messages can be categorized based on the type of message (results, medications, schedule, and general) and interpreted in the context of the patient’s medical record [56]. New information can be sent to the electronic health record through an API call. Agent-suggested changes to the medical record (medication history, medical history, compliance, symptoms, or diagnosis) can be updated with a provider’s approval. At the same time, a relevant reply can be drafted or sent automatically to guide a patient to the next step in their care plan.

#### Medical History Agents

Medical history agents describe AI tools that modernize and streamline the history-taking process for patients and provide thorough and relevant information as necessary to providers [57]. These chatbots present patients with targeted, personalized questions, providing an accessible and attractive option for history taking [57,58]. They specialize in organizing relevant information for providers to deliver a summary that can quickly prepare a provider for a clinical encounter [54]. These agents are essential for the functions of an agentic health care practice, providing relevant information as needed for both caretakers and other agents.

In a systematic review of studies on AI chatbots, Hindelang et al [57] review the uses of different chatbots in clinical settings. A study on a medical history bot used to support anamnesis could reduce time spent on history in patient encounters by over 50% [59]. Another example of a medical history agent is AnCha, an AI interviewer that supports the collection of history and current complaints for a patient. Previsit documentation of relevant medical history could prime patients for clinical encounters and provide information for upstream use where higher priority items are given more emphasis to reduce visit time. Future versions of AnCha or similar bots could solve language barriers or present questions with images that patients can interact with, further increasing accessibility and efficacy of history taking [54,57,60]. The unique option for providers to let patients know an AI agent is being used may further remove stigma or hesitation from patients due to less fear of being judged or “labeled.” In urologic practice, previsit agents could systematically capture smoking history, occupational exposures, and anticoagulant use for hematuria evaluations; gonadotoxin and surgical history for male infertility consultations; and International Prostate Symptom Score and prior procedural history for Benign prostatic hyperplasia BPH assessments, reducing intake time while improving completeness.

#### Triage Agents

Triage agents are designed to automate or support staff in determining the urgency and priority of care to appropriately designate resources based on a patient’s history and current status. They can work with medical history agents to review patient backgrounds, symptoms, and vital signs to assign triage categories or recommend care plans. They are essential as a prerequisite for further care delivery, decision support, or patient-facing digital assistants that collect patient histories [46,54,61-64]. They have demonstrated efficacy and efficiency in rapidly triaging patients, reducing wastefulness of resources, and minimizing undertriaging and overtriaging [61].

In 1 study, a novel triage prediction AI model was trained to analyze prior patient data retrospectively to determine if it could more precisely and accurately determine potential health outcomes. Emergency room physicians were asked to predict the criticality of 30 visits extracted from a dataset of 171,275. The AI model outperformed physicians in both categories in determining criticality of health outcomes, with a sensitivity of 95% compared to physicians’ 41%, and an accuracy of 90% compared to physicians’ 67%. While the study was based on retrospective data in China and lacked a validation cohort, it demonstrates the potential of predictive analysis in AI tools [65]. In a similar separate study, researchers trained an AI model to review patient medical information to provide an accurate Emergency Severity Index (ESI) value. Nurses were asked to assign an ESI value from 800 samples from a dataset of 147,052. The model estimated with an accuracy of 75.9%, compared to nurses with 59.8% and study clinicians with 75.3%, with fewer errors and more accurate high-risk recognition [66]. Both studies were prospective and lacked a validation cohort, which questions their efficacy in other clinical settings. However, both studies demonstrate the potential for triage agents to efficiently and accurately assess patient priority, reducing the burden on clinicians, increasing accuracy of triage, and reducing costs through effective resource allocation. While this approach does raise questions about scope of practice and triage, which is regulated in many states, it allows for a first pass of information, though it will require some medical-legal input to determine who ultimately holds the responsibility of inappropriate triage. In urology, triage agents could differentiate obstructing infected ureteral stones from uncomplicated colic based on fever, urinalysis, and symptom pattern, and could screen postoperative patients following endoscopic or robotic procedures for red-flag symptoms warranting urgent provider notification.

#### Decision Support Agents

Decision support agents can assist in the diagnosis and treatment of complex or rare clinical conditions based on clinical guidelines and contribute to multidisciplinary patient case management. They can help establish personalized treatment plans and act as a resource to physicians in increasing efficiency and accuracy of care delivery [67]. By analyzing large volumes of health data, these agents can evaluate risk predictions, provide diagnostic support, and adapt to new information in more complex clinical situations [67]. This has allowed for the development of AI tools that provide advice for clinical decision-making, indicative of future health care agents that can assist physicians through clinical reasoning, acting as a well-versed peer to support personalized patient care [68].

AI-based tools have been used in numerous clinical situations for predictive or early diagnostic support [21,69-71]. In urologic settings, AI tools have applications for prostate brachytherapy, biopsy, cancer diagnosis, and cancer staging [72-74]. They have demonstrated the ability to analyze and grade radiomics features of renal cell carcinoma patients with comparable performance to renal biopsy, reducing the need for invasive alternatives [47]. Furthermore, they have demonstrated efficacy in estimating the response of a bladder cancer lesion to chemotherapy, matching or outperforming estimations of radiologists [75]. These agents may also be able to look at chemotherapeutic options for patients by analyzing pathology and cytology in the setting of clinical studies and case reports, as well as cancer recurrence predictions.

#### Documentation Agents

Documentation agents support health care providers by capturing audio in clinical settings, transcribing text, and generating drafts of clinical notes for physician review [76-78]. These agents can dramatically reduce the amount of time providers spend on documentation, allowing providers to be more engaged in patient conversation [76]. These agents could further surpass the capabilities of traditional LLMs by placing orders with proper billing and coding in place for physicians to review [22]. In a space where documentation burdens directly contribute to provider burnout, documentation agents can fill a critical need for providers to improve care delivery and health outcomes [79].

Amongst the more than 90 ambient scribing platforms that exist at the time of this article’s publishing, 1 study reviewed DAX-copilot, an AI-powered clinical documentation agent owned by Microsoft [76,78]. Researchers were interested in the potential benefits of the tool, the types of encounters fitting for AI technology, and physician impressions on AI-facilitated clinical documentation. The study found that providers saw a significant reduction in time spent on clinical documentation and cognitive tasks, allowing for more time to be spent on engaged care delivery. While the providers had some hesitance towards using the AI agent in some situations, and documentation notation occasionally fell short of standards, physicians recognized the potential for the tool to improve their quality of life, reduce their daily cognitive burden, and improve quality of engagement with patients [76]. Microsurgical operative reports for procedures such as varicocelectomy, vasectomy reversal, and spermatic cord denervation require detailed anatomic narrative and current procedural terminology (CPT) justification. Documentation agents could generate structured drafts from intraoperative dictation, reducing post-case charting burden and improving coding accuracy.

Amongst the more than 90 ambient scribing platforms that exist at the time of this article’s publishing, 1 study reviewed DAX-copilot, an AI-powered clinical documentation agent owned by Microsoft [76,78]. Researchers were interested in the potential benefits of the tool, the types of encounters fitting for AI technology, and physician impressions on AI-facilitated clinical documentation. The study found that providers saw a significant reduction in time spent on clinical documentation and cognitive tasks, allowing for more time to be spent on engaged care delivery. While the providers had some hesitance towards using the AI agent in some situations, and documentation notation occasionally fell short of standards, physicians recognized the potential for the tool to improve their quality of life, reduce their daily cognitive burden, and improve quality of engagement with patients [76]. Microsurgical operative reports for procedures such as varicocelectomy, vasectomy reversal, and spermatic cord denervation require detailed anatomic narrative and CPT justification. Documentation agents could generate structured drafts from intraoperative dictation, reducing post-case charting burden and improving coding accuracy.

#### Care Navigation Agent

Care navigation describes guiding patient progress throughout their larger health care journey, ensuring that transitions between different aspects of care are seamless and continuous. Care navigation agents support patients and assist caretakers in adequate preparation for care delivery, navigating follow-ups, and ensuring positive outcomes [67,80]. They can schedule appointments or referrals and share information with relevant providers to ensure that patients have proper follow-up with positive outcomes [14,80]. As conversational agents or chatbots, they can provide greater accessibility to patients with limited access to in-person care while maintaining a personalized experience, allowing for caretakers to support larger populations of patients more effectively [14]. They can consider the context of a patient’s environment or social determinants of health to identify social needs [14,81].

One study used a care navigation agent aware of a patient’s clinical, socioeconomic, and behavioral data to predict the likelihood of patient readmission and provide care navigators with tips to prevent rehospitalization [82]. Out of over 6000 encounters, the AI identified 29.3% as medium to high risk for rehospitalization within 30 days, and provided recommendations for care navigators. Of the high-risk adults provided recommendations, the navigation team saw a 21% decrease in incidence of 30-day rehospitalization, or 69 fewer rehospitalizations for every 1000 encounters [82]. With a statistically significant sample size, the study demonstrates the capabilities of care navigation agents to reduce health care costs by minimizing unnecessary rehospitalizations while improving transitional care outcomes. Care navigation agents may also be able to provide resources to address social determinants of health, including financial support for medications, utilities, as well as links to various social networking options and support groups that may be a good fit for an individual [81,83]. Nonmuscle invasive bladder cancer surveillance, which requires cystoscopy and cytology at defined intervals over years, is an ideal navigation use case. Agents could manage scheduling, coordinate pathology review, and alert providers to findings meeting criteria for disease progression or treatment escalation.

#### Health and Wellness Coaching Agent

Health and wellness coaching agents provide personalized, holistic, interactive care through chatbots or virtual assistants to support lifestyle behavior change for patients coping with chronic issues outside of the hospital. These AI agents read patient data to deliver a tailored experience through personalized goal setting, real-time feedback, motivational support, medical education on lifestyle and behavioral changes, and stress management. By providing consistent availability for patients automatically, or by supporting human coaches with suggestions and quality control, these agents provide scalable, accessible, and on-demand services through virtual support platforms [84-86].

A multitude of health and wellness coaching AI support agents exist in the current health care market, such as Tess (X2AI Inc), Lark (Lark Health), and Woebot (Woebot Health), conversational AI that support patients in lifestyle and behavior change [67,87-89]. These agents have demonstrated the ability to drive engagement with care plans and healthier lifestyle behaviors to reach and maintain health goals reviewed by providers. One study specifically examined how a virtual chatbot, Paola (University of South Australia), could support patients in increasing their exercise, maintenance to a Mediterranean diet, and losing weight [87]. Compared to the baseline, these patients completed 109 more minutes of exercise after 12 wk, lost 1.3 more kg of weight, and lost 2.1 cm from their waistline [87]. Increased health and wellness coaching services are already being used at scale to support patient care outside of the clinic, and these agents demonstrate the potential to help patients succeed in reaching goals in an accessible, low-cost, and scalable process. Obesity, metabolic syndrome, and physical inactivity are modifiable risk factors for erectile dysfunction and hypogonadism (Hehemann and Kashanian 2016). Coaching agents could deliver structured behavioral interventions targeting these factors between visits, reinforcing lifestyle changes that complement pharmacologic or hormonal treatment.

#### Patient Monitoring Agents

One of the challenges in remote patient monitoring is the amount of incoming data and the ability to appropriately spend time reviewing and synthesizing the various data inputs in a meaningful way for clinical decision-making. Ongoing patient monitoring post care delivery is essential for quality control, quality improvement, and ensuring safety of interventions. To meet the need for this quality assurance, patient monitoring agents can leverage ongoing changes in patient data from electronic health records, wearable devices, and digital sources to recognize deviations in expected outcomes, poor adherence to treatment recommendations, safety issues, and support quality improvement [90,91]. Patient monitoring agents can be used to predict when changes in patient health status increase risk, alerting caretakers or other agents when interventions may be necessary [92]. As AI algorithms become more widely used in health care settings, traditional methods of quality and outcome monitoring may not be able to maintain scalability or allow for wider testing of intervention strategies [48,90]. Outcome monitoring agents could be effective support resources that ensure scalable health care delivery systems are continually delivering quality care, and that interventions are effective and safe while upholding ethical and clinical standards [93].

One study proposed the use of AI as a tool for both reliably communicating and effectively predicting changes in vital signs of a critical patient [91]. The study found that an AI-powered tool was able to effectively decrease the probability of communication failures between monitoring devices and alarm systems. Furthermore, they demonstrated that the tool was capable of predicting both normal and critical fluctuations in data from patient monitoring devices in as little as 25 ms. AI tools such as these demonstrate the capability to improve upon current remote and local patient monitoring processes, as well as enhancing other aspects of the agentic care delivery system by rapidly predicting changes in patient status [91]. This information can be shared with care navigation agents and health and wellness coaching agents to ensure proper and effective follow-up. Following radical prostatectomy, monitoring agents could track patient-reported catheter output and hematuria. During androgen deprivation therapy, agents could survey for metabolic complications including glycemic changes and weight gain, alerting providers when intervention thresholds are met.

#### Administrative Agents

Administrative agents can automate or augment the nonclinical, operational, or organizational tasks within health care systems to increase provider efficiency [30,94,95]. They can support nurses in streamlining processes such as patient admissions, transfers, and discharges, saving 37%‐46% of time spent on those tasks [94]. They further can improve the efficiency of billing processes by expediting the reimbursement process, ensuring proper coding, detecting fraud, and even revenue optimization [30,96,97]. Administrative AIs have shown to reduce coding errors and manual workload, improving efficiency and mitigating financial losses [97,98]. While LLMs alone have the potential to expedite 15% of labor tasks, tool-enhanced LLMs like administrative agents increase that percentage to over 46% [99].

A study discussed how the time-intensive and high-pressure environment of the operating room increases the chances of errors in coding, a burden that could be relieved by AI [100]. Researchers implemented an AI model to extract procedure codes from free-text surgical notes to provide a transparent and standardized coding validation solution. In over 3000 reviewed cases with over 8000 CPT codes, the model was able to identify errors in 12% of cases and outperformed surgeons in adherence to the referenced standard. Furthermore, prospective testing on 268 notes demonstrated effective and applicable real-world performance. Studies such as these demonstrate real-world applications for AI solutions that enhance providers' ability to deliver care while promoting cost-saving and efficiency. Complex urologic procedures involving layered CPT coding, such as microsurgical varicocelectomy with concurrent denervation or multicomponent penile prosthetic revision, are high-value targets for administrative agents that can reduce undercoding and generate payer-specific prior authorization documentation.

#### Research Agent

Research agents are autonomous systems that sense and interpret information from large datasets to support clinicians in care delivery, or in assisting scientific researchers in conducting effective and efficient research [20,101]. They can function as chatbots that support providers by leveraging patient data to explore external research relevant to care delivery, or as researchers themselves that adapt to biological insights, incorporate new scientific findings, and refine hypotheses as necessary in the research process [101,102]. As current research agents struggle to develop novel hypotheses, these agents mostly improve upon current research techniques by finding more refined parameters to improve upon baselines [103]. However, research agents have been used to understand the structure and function of protein sequences and genes, analyze phenotypes, and review clinical outcomes through the analysis of biomedical data [103-106]. Research agents could be a vital component of future health care establishments that aim to be driving scientific understanding while providing effective and relevant care. Open Evidence (OpenEvidence Inc.) is an example of a tool many providers are using to query high-quality clinical journals to identify answers to clinical questions. Incorporating such tools into an electronic medical record to be able to query sources of data based on individual patient information can provide real-time support for providers [81].

### An Agentic Health Care System

#### Overview

Current shortcomings in health care delivery necessitate innovation that can drive care delivery systems towards healthcare 5.0. AI is already being incorporated throughout different domains of health care to achieve this aim. Fahim et al describes AI-powered innovations ranging from personalized therapies that improve patient responsiveness and care plan compliance to smart-alerts that notify providers when critical levels appear in monitoring devices [107]. Alowais et al highlight AI’s capabilities to predict drug responses based on patient history or reduce administrative notetaking burdens of providers [15]. AI agents that incorporate these features could be the next step in improving accessibility and driving personalized, value-based care without compromising cost or quality of care. The agents described above could represent a future health care practice that effectively uses resources and available technology to meet the growing demands of a changing health care landscape.

#### Proposed Operational Framework

To better describe the operations of AI agents, we describe the following conceptual framework. Patient health information remains at the center, protected by Health Insurance Portability and Accountability Act compliant and secure records barriers. Immediately surrounding protected health information are trusted, similarly secure, and minimally probability-based machine learning models that can recognize and categorize data for specialized purposes. Additional layers of security for protecting patient information stand between this categorized data output and Health Insurance Portability and Accountability Act compliant LLMs which communicate directly with the AI agents we describe. The agents are experts in processing data tailored to their specific function, are provider and patient facing, and require less security to access. Safety and security checks are present at all levels of iteration.

## Bridging the Future of Men’s Health Urology to Today

The future of men’s health care depends on our ability to adapt to changing technology while maintaining ethical standards. In embracing innovation, future systems that achieve the goals of the quintuple aim are within sight.

### A Vision of the Patient Experience in a Healthcare 5.0 Urology Clinic

**Figure 3.**
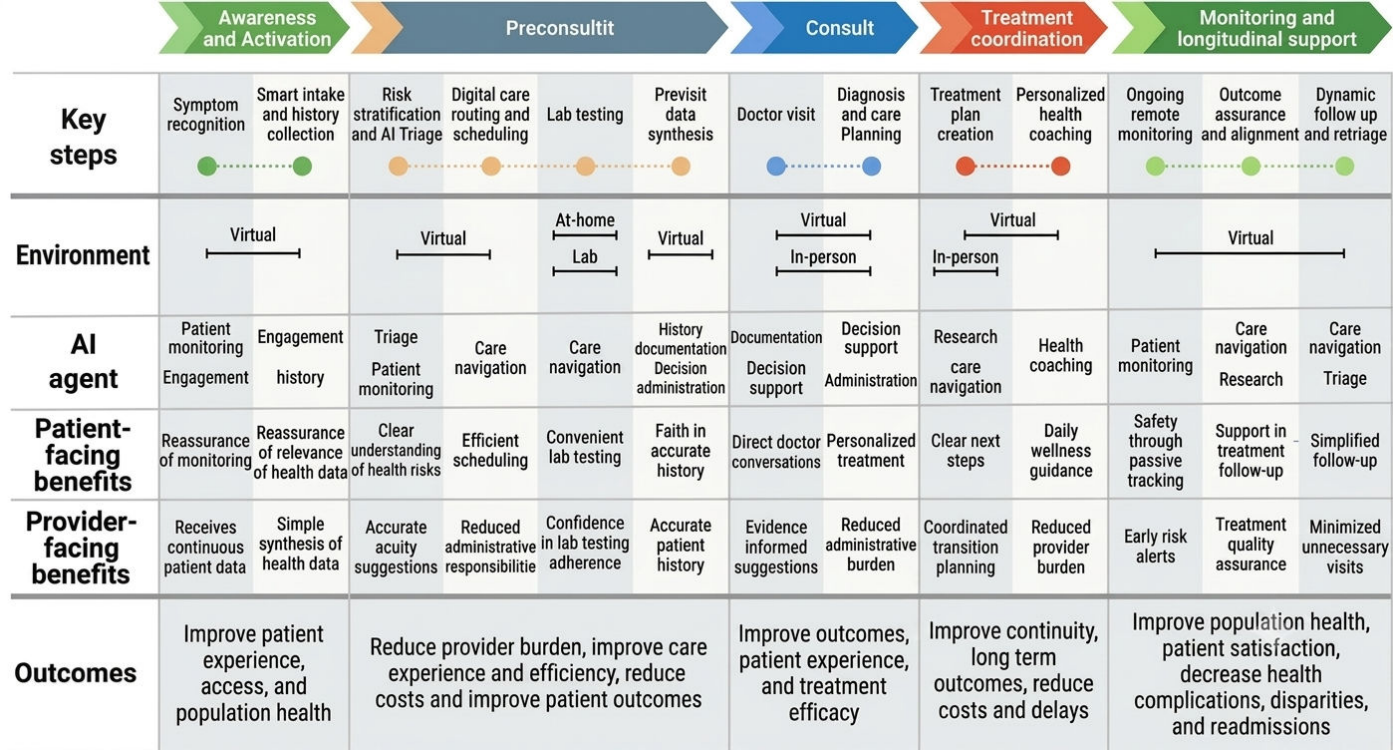
A workflow proposing the key steps in the patient journey of a Healthcare 5.0 urology clinic. The proposed key steps in each phase of the journey are described along with the agentic tools relevant to each step, the benefits for providers and patients, and the larger health care outcomes contributed to.

A Healthcare 5.0 urology clinic of the future provides a seamless, personalized, and optimized journey from start to finish, depicted in Figure 3. A patient experiencing new urologic symptoms initiates the onboarding process for virtual care via a mobile app. An engagement agent provides personalized and intriguing questions, referencing a medical history agent’s presentation of relevant prior data. A triage agent synthesizes the patient’s presentation and history to determine patient acuity, while the engagement agent shares relevant educational information based on symptoms. A care navigation then shares the relevant information with the patient’s care team, working with staff to optimally schedule their laboratory visits and consultations. After completing further laboratory or at-home tests, the patient’s relevant data is collected, summarized, and presented to the patient’s physician to deliver a relevant history. Based on complexity, the patient completes an in-person or virtual consultation. Documentation agents, research agents, and decision support agents support a urologist in providing efficient, human-centric care, equipping a provider to focus on the patient while capturing key information and providing evidence-based recommendations in real time. Administrative agents work with nurses, care navigation agents, and documentation agents to prepare the patient’s next steps based on an optimized treatment and scheduling plan while the patient is completing their visit. Appropriate orders have been curated so that the urologist merely reviews and signs off on them, or at a higher level, uses appropriate voice recognition or biometrics as a signature pattern instead. After the consult, health coaching agents ensure the patient receives proper personalized support, encouraging lifestyle changes and adherence to prescriptions that improve outcomes. Behind the scenes, administrative agents assist providers in remaining compliant with hospital policies and ethical standards while providing seamless billing and coding support, and research agents monitor intervention efficacy while supporting provider research. Patient monitoring and care navigation agents provide active surveillance of patient health status, ensuring that any changes or sudden increases in risk are recorded and reported to caretakers as needed. A cohesive interaction between technology, providers, and patients creates a proactive, intelligent, and human-centered specialty care experience.

### Future Directions

To bridge the promise of AI-driven agentic systems and further technological innovations with the urgent realities facing men’s health urology today, future efforts must tackle specific gaps and drive robust, sustainable adoption at scale.

#### Build and Validate the Virtual-First Model

Future research should develop and rigorously test virtual-first urology care models that handle the majority of cases through AI-enabled telehealth, reserving brick-and-mortar encounters for only the most complex needs. Prospective studies should measure safety, patient outcomes, provider satisfaction, and cost-effectiveness compared to traditional episodic, in-person models [108,109]. However, issues intrinsic to machine learning models, like algorithmic bias and brittleness, present challenges to implementation currently. Furthermore, costs of implementing AI systems at scale, limited datasets, and privacy concerns are variables that must be addressed prior to implementation [110].

A critical limitation of this paper is that we discuss agentic AI as a unified concept without addressing condition-specific accuracy requirements. Different urologic conditions require different performance thresholds: low-acuity conditions (urinary tract infection screening) can tolerate higher error rates than high-acuity conditions (cancer staging). Rigorous implementation will require condition-specific validation protocols that are beyond the scope of this conceptual framework. Furthermore, performance thresholds must be influenced by risk-stratified deployment protocols tied to the consequence of clinical error. Areas that require human oversight must be clearly defined, and prospective studies must be validated in an applicable patient population. The limitations on our paper represent important areas for future prospective clinical research. Real-world implementation must depend on prospective validation, safety monitoring, bias assessment, regulatory compliance, and clinician oversight.

#### Develop Interoperable Data Ecosystems

Scalable agentic systems rely on seamless data integration across electronic health records, wearables, remote monitoring devices, and AI modules. Future work must address data standardization, secure interoperability, and patient-controlled consent frameworks to enable continuous learning and context-aware decision-making while safeguarding privacy [108,109]. It is essential that AI agents ensure that correct critical information is passed without bias. Using Fast Healthcare Interoperability Resources standards in the initial development of AI agents will allow for effective and more seamless integration into current health records and clinical systems.

#### Design for Real-World Workflow Integration

Successful transformation will depend on aligning AI agents with the daily workflows of providers and support staff. Implementation science should guide the design of interfaces, training, and human-AI comanagement protocols that minimize friction, reduce cognitive burden, and promote trust among both clinicians and patients [108,111,112].

#### Strengthen Evidence for Agentic Roles

While early demonstrations are promising, from triage accuracy to ambient scribing, large-scale, condition-specific validation is needed, especially in urology. Future research should further clarify how the AI agents described previously translate to measurable improvements in outcomes and health equity [108,112].

#### Address Regulatory, Ethical, and Reimbursement Barriers

As AI systems assume more complex, autonomous functions, policy frameworks must evolve to clarify liability, algorithmic transparency, and ethical use. Research should inform adaptive regulations and reimbursement models that incentivize virtual-first practices while ensuring patient safety and equitable access [109]. In addition, legal input will be required for specific agents whose roles may have some degree of overlap with a licensed role, such as a registered nurse and triage, and in some cases, a health coach based on individual state statutes.

Especially during initial implementations, human involvement in key steps of the implementation of AI systems is necessary for long-term practical use [113]. Clinicians provide necessary guidance that can ensure reasonable systems are introduced, that regulate care delivery methods, and maintain quality of care over time [114]. For example, in a clinic looking to introduce agentic AI systems, clinicians could review actions proposed by the agent and check off on actions prior to execution. Actions performed autonomously by the agents can be monitored and flagged for review, allowing for quality monitoring over time. Practical human-in-the-loop frameworks such as this promote feasibility, improve provider trust in AI systems, and encourage patient safety.

#### Embed Equity and Inclusivity by Design

Future work must prioritize inclusive training data and bias auditing to ensure AI agents perform equitably across diverse populations. Studies should test deployment in underserved communities, rural regions, and linguistically diverse groups, using community partnerships to design culturally competent agentic interactions [111].

#### Enable Continuous Learning and Improvement

The promise of Healthcare 5.0 depends on robust feedback loops. Future directions should include real-world monitoring, outcome auditing, and rapid iteration of AI models to adapt to evolving evidence, changing guidelines, and patient needs — ensuring that innovations remain clinically relevant and safe [109].

### Conclusion

By addressing these directions, the integration of telehealth and agentic AI can advance from a promising framework to an operational reality, positioning men’s health urology at the forefront of equitable, efficient, and scalable care for the decade ahead.

## Abbreviations

CPT: current procedural terminology
ESI: Emergency Severity Index
LLM: large language model

## References

[1] Okeke CJ, Jeje EA, Obi AO, Ojewola RW, Ogbobe UU (2023). The burden of urologic diseases in a tertiary hospital in South-eastern Nigeria: a three-year review. J West Afr Coll Surg.

[2] Miller DC, Saigal CS, Litwin MS (2009). The demographic burden of urologic diseases in America. Urol Clin North Am.

[3] Cleveland clinic survey reveals men’s top health concerns as they age. Cleveland Clinic.

[4] Our priority: address the urologic workforce shortage. American Urological Association.

[5] Healthcare costs associated with first-line (1L) treatment of patients with locally advanced or metastatic urothelial carcinoma (la/muc) in the united states (US). American Society of Clinical Oncology.

[6] Avritscher EBC, Cooksley CD, Grossman HB (2006). Clinical model of lifetime cost of treating bladder cancer and associated complications. Urology.

[7] Joyce DD, Sharma V, Jiang DH (2022). Out-of-pocket cost burden associated with contemporary management of advanced prostate cancer among commercially insured patients. J Urol.

[8] Zhu C, Wang DQ, Zi H (2021). Epidemiological trends of urinary tract infections, urolithiasis and benign prostatic hyperplasia in 203 countries and territories from 1990 to 2019. Mil Med Res.

[9] Bray F, Laversanne M, Sung H (2024). Global cancer statistics 2022: GLOBOCAN estimates of incidence and mortality worldwide for 36 cancers in 185 countries. CA Cancer J Clin.

[10] Farrell TW, Greer AG, Bennie S, Hageman H, Pfeifle A (2023). Academic health centers and the quintuple aim of health care. Acad Med.

[11] Shah YB, Goldberg ZN, Harness ED, Nash DB (2024). Charting a path to the quintuple aim: harnessing AI to address social determinants of health. Int J Environ Res Public Health.

[12] Yu C, Xian Y, Jing T (2023). More patient-centered care, better healthcare: the association between patient-centered care and healthcare outcomes in inpatients. Front Public Health.

[13] Sacks GD, Lawson EH, Dawes AJ (2015). Relationship between hospital performance on a patient satisfaction survey and surgical quality. JAMA Surg.

[14] Li Y, Liang S, Zhu B (2023). Feasibility and effectiveness of artificial intelligence-driven conversational agents in healthcare interventions: a systematic review of randomized controlled trials. Int J Nurs Stud.

[15] Alowais SA, Alghamdi SS, Alsuhebany N (2023). Revolutionizing healthcare: the role of artificial intelligence in clinical practice. BMC Med Educ.

[16] Baldwin K, Ginsberg P, Harkaway RC (2003). Under-reporting of erectile dysfunction among men with unrelated urologic conditions. Int J Impot Res.

[17] Clark H, Venishetty N, Howell S, Deebel NA, Muthigi A (2026). Utilization of artificial intelligence in men’s health: opportunities for innovation and quality improvement. Int J Impot Res.

[18] Meder B, Asselbergs FW, Ashley E (2025). Artificial intelligence to improve cardiovascular population health. Eur Heart J.

[19] Sahni NR, Carrus B (2023). Artificial intelligence in U.S. health care delivery. N Engl J Med.

[20] Singh K, Prabhu A, Kaur N (2025). The impact and role of artificial intelligence (AI) in healthcare: systematic review. Curr Top Med Chem.

[21] Loftus TJ, Shickel B, Ozrazgat-Baslanti T (2022). Artificial intelligence-enabled decision support in nephrology. Nat Rev Nephrol.

[22] Qiu J, Lam K, Li G (2024). LLM-based agentic systems in medicine and healthcare. Nat Mach Intell.

[23] Luo W, Nguyen T, Nichols M (2015). Is demography destiny? application of machine learning techniques to accurately predict population health outcomes from a minimal demographic dataset. PLoS ONE.

[24] Shaban-Nejad A, Michalowski M, Buckeridge DL (2018). Health intelligence: how artificial intelligence transforms population and personalized health. NPJ Digital Med.

[25] Shrank WH, Rogstad TL, Parekh N (2019). Waste in the US health care system: estimated costs and potential for savings. JAMA.

[26] Maciosek MV, Coffield AB, Flottemesch TJ, Edwards NM, Solberg LI (2010). Greater use of preventive services In U.S. health care could save lives at little or no cost. Health Aff (Millwood).

[27] Dehmer SP, Maciosek MV, LaFrance AB, Flottemesch TJ (2017). Health benefits and cost-effectiveness of asymptomatic screening for hypertension and high cholesterol and aspirin counseling for primary prevention. Ann Fam Med.

[28] Wolff J, Pauling J, Keck A, Baumbach J (2020). The economic impact of artificial intelligence in health care: systematic review. J Med Internet Res.

[29] Gomez Rossi J, Rojas-Perilla N, Krois J, Schwendicke F (2022). Cost-effectiveness of artificial intelligence as a decision-support system applied to the detection and grading of melanoma, dental caries, and diabetic retinopathy. JAMA Netw Open.

[30] Spear J, Ehrenfeld JM, Miller BJ (2023). Applications of artificial intelligence in health care delivery. J Med Syst.

[31] Vithlani J, Hawksworth C, Elvidge J, Ayiku L, Dawoud D (2023). Economic evaluations of artificial intelligence-based healthcare interventions: a systematic literature review of best practices in their conduct and reporting. Front Pharmacol.

[32] Sahni N, Stein G, Zemmel R, Cutler D (2023). The potential impact of artificial intelligence on healthcare spending.

[33] Kumar D, Malin BA, Vishwanatha JK, Wu L, Hedges JR (2024). AI in biomedicine-a forward-looking perspective on health equity. Int J Environ Res Public Health.

[34] Clark CR, Wilkins CH, Rodriguez JA (2021). Health care equity in the use of advanced analytics and artificial intelligence technologies in primary care. J Gen Intern Med.

[35] Ghanem S, Moraleja M, Gravesande D, Rooney J (2025). Integrating health equity in artificial intelligence for public health in Canada: a rapid narrative review. Front Public Health.

[36] Chin MH, Afsar-Manesh N, Bierman AS (2023). Guiding principles to address the impact of algorithm bias on racial and ethnic disparities in health and health care. JAMA Netw Open.

[37] Budhwani S, Fujioka J, Thomas-Jacques T (2022). Challenges and strategies for promoting health equity in virtual care: findings and policy directions from a scoping review of reviews. J Am Med Inform Assoc.

[38] Mizna S, Arora S, Saluja P, Das G, Alanesi WA (2025). An analytic research and review of the literature on practice of artificial intelligence in healthcare. Eur J Med Res.

[39] Topol EJ (2019). High-performance medicine: the convergence of human and artificial intelligence. Nat Med.

[40] Osama M, Ateya AA, Sayed MS (2023). Internet of medical things and healthcare 4.0: trends, requirements, challenges, and research directions. Sensors (Basel).

[41] Tandel V, Kumari A, Tanwar S, Singh A, Sharma R, Yamsani N (2024). Intelligent wearable-assisted digital healthcare industry 5.0. Artif Intell Med.

[42] Bisset B, Shriram T, Davuluri M (2025). Applications and outcomes of telehealth and integrated care in men’s health urology. J Med Internet Res.

[43] Sharma N (2026). Healthcare 50: AI Driven Workspace in Sustainable Telehealth.

[44] Basulo-Ribeiro J, Teixeira L (2024). The future of healthcare with industry 5.0: preliminary interview-based qualitative analysis. Future Internet.

[45] Yuan H (2025). Agentic large language models for healthcare: current progress and future opportunities. Med Adv.

[46] Arslan B, Nuhoglu C, Satici MO, Altinbilek E (2025). Evaluating LLM-based generative AI tools in emergency triage: a comparative study of ChatGPT Plus, Copilot Pro, and triage nurses. Am J Emerg Med.

[47] Suarez-Ibarrola R, Hein S, Reis G, Gratzke C, Miernik A (2020). Current and future applications of machine and deep learning in urology: a review of the literature on urolithiasis, renal cell carcinoma, and bladder and prostate cancer. World J Urol.

[48] Moazemi S, Vahdati S, Li J (2023). Artificial intelligence for clinical decision support for monitoring patients in cardiovascular ICUs: a systematic review. Front Med.

[49] Angus DC, Khera R, Lieu T (2025). AI, health, and health care today and tomorrow. JAMA.

[50] Bajwa J, Munir U, Nori A, Williams B (2021). Artificial intelligence in healthcare: transforming the practice of medicine. Future Healthc J.

[51] Karunanayake N (2025). Next-generation agentic AI for transforming healthcare. Inform Health.

[52] Wah JNK (2025). Revolutionizing e-health: the transformative role of AI-powered hybrid chatbots in healthcare solutions. Front Public Health.

[53] Genovese A, Borna S, Gomez-Cabello CA (2024). Artificial intelligence in clinical settings: a systematic review of its role in language translation and interpretation. Ann Transl Med.

[54] Chaix B, Bibault JE, Pienkowski A (2019). When chatbots meet patients: one-year prospective study of conversations between patients with breast cancer and a chatbot. JMIR Cancer.

[55] Tersoo Catherine A, Towfek SK, A. Abdelhamid A (2023). An overview of the evolution and impact of chatbots in modern healthcare services. Mesopotamian J Artif Intell Healthc.

[56] Garcia P, Ma SP, Shah S (2024). Artificial intelligence–generated draft replies to patient inbox messages. JAMA Netw Open.

[57] Hindelang M, Sitaru S, Zink A (2024). Transforming health care through chatbots for medical history-taking and future directions: comprehensive systematic review. JMIR Med Inform.

[58] Hong G, Smith M, Lin S (2022). The AI will see you now: feasibility and acceptability of a conversational AI medical interviewing system. JMIR Form Res.

[59] Schneider S, Gasteiger C, Wecker H (2023). Successful usage of a chatbot to standardize and automate history taking in hymenoptera venom allergy. Allergy.

[60] Gashi F, Regli SF, May R, Tschopp P, Denecke K (2021). Developing intelligent interviewers to collect the medical history: lessons learned and guidelines. Stud Health Technol Inform.

[61] Da’Costa A, Teke J, Origbo JE, Osonuga A, Egbon E, Olawade DB (2025). AI-driven triage in emergency departments: a review of benefits, challenges, and future directions. Int J Med Inform.

[62] Defilippo A, Veltri P, Lió P, Guzzi PH (2024). Leveraging graph neural networks for supporting automatic triage of patients. Sci Rep.

[63] Siira E, Johansson H, Nygren J (2025). Mapping and summarizing the research on AI systems for automating medical history taking and triage: scoping review. J Med Internet Res.

[64] Kachman MM, Brennan I, Oskvarek JJ, Waseem T, Pines JM (2024). How artificial intelligence could transform emergency care. Am J Emerg Med.

[65] Chen MC, Huang TY, Chen TY, Boonyarat P, Chang YC (2023). Clinical narrative-aware deep neural network for emergency department critical outcome prediction. J Biomed Inform.

[66] Ivanov O, Wolf L, Brecher D (2021). Improving ED emergency severity index acuity assignment using machine learning and clinical natural language processing. J Emerg Nurs.

[67] Milne-Ives M, de Cock C, Lim E (2020). The effectiveness of artificial intelligence conversational agents in health care: systematic review. J Med Internet Res.

[68] van Baalen S, Boon M, Verhoef P (2021). From clinical decision support to clinical reasoning support systems. J Eval Clin Pract.

[69] Wu M, Du X, Gu R, Wei J (2021). Artificial intelligence for clinical decision support in sepsis. Front Med.

[70] Bizzo BC, Almeida RR, Michalski MH, Alkasab TK (2019). Artificial intelligence and clinical decision support for radiologists and referring providers. J Am Coll Radiol.

[71] Parsi A, Glavin M, Jones E, Byrne D (2021). Prediction of paroxysmal atrial fibrillation using new heart rate variability features. Comput Biol Med.

[72] Garapati SS, Hadjiiski L, Cha KH (2017). Urinary bladder cancer staging in CT urography using machine learning. Med Phys.

[73] Nouranian S, Ramezani M, Spadinger I, Morris WJ, Salcudean SE, Abolmaesumi P (2016). Learning-based multi-label segmentation of transrectal ultrasound images for prostate brachytherapy. IEEE Trans Med Imaging.

[74] Jiang Y, Yang M, Wang S, Li X, Sun Y (2020). Emerging role of deep learning‐based artificial intelligence in tumor pathology. Cancer Commun (Lond).

[75] Wu E, Hadjiiski LM, Samala RK (2019). Deep learning approach for assessment of bladder cancer treatment response. Tomography.

[76] Bundy H, Gerhart J, Baek S (2024). Can the administrative loads of physicians be alleviated by AI-facilitated clinical documentation?. J Gen Intern Med.

[77] Doshi GK, Jensen TL, Graziano A, Enenmoh C, Lindsey J (2024). Use of ambient AI scribing: impact on physician administrative burden and patient care. JCO Oncol Pract.

[78] Kunze KN, Bepple J, Bedi A, Ramkumar PN, Pean CA (2025). Commercial products using generative artificial intelligence include ambient scribes, automated documentation and scheduling, revenue cycle management, patient engagement and education, and prior authorization platforms. Arthroscopy.

[79] Bracken A, Reilly C, Feeley A, Sheehan E, Merghani K, Feeley I (2025). Artificial intelligence (AI) - powered documentation systems in healthcare: a systematic review. J Med Syst.

[80] Tudor Car L, Dhinagaran DA, Kyaw BM (2020). Conversational agents in health care: scoping review and conceptual analysis. J Med Internet Res.

[81] Davis VH, Pinto AD, Patel MR (2025). Leveraging artificial intelligence to inform care coordination by identifying and intervening in patients’ unmet social needs: a scoping review. J Adv Nurs.

[82] Brown Z, Bergman D, Holt L (2023). Augmenting a transitional care model with artificial intelligence decreased readmissions. J Am Med Dir Assoc.

[83] Matulis J, McCoy R (2023). Relief in sight? chatbots, in-baskets, and the overwhelmed primary care clinician. J Gen Intern Med.

[84] Bojic I, Ong QC, Ito S (2025). AI-empowered health coaching for university students: a mixed-method process evaluation. Comput Biol Med.

[85] Fadhil A, Wang Y, Reiterer H (2019). Assistive conversational agent for health coaching: a validation study. Methods Inf Med.

[86] Terblanche N, Molyn J, de Haan E, Nilsson VO (2022). Comparing artificial intelligence and human coaching goal attainment efficacy. PLoS One.

[87] Maher CA, Davis CR, Curtis RG, Short CE, Murphy KJ (2020). A physical activity and diet program delivered by artificially intelligent virtual health coach: proof-of-concept study. JMIR Mhealth Uhealth.

[88] Aggarwal A, Tam CC, Wu D, Li X, Qiao S (2023). Artificial intelligence-based chatbots for promoting health behavioral changes: systematic review. J Med Internet Res.

[89] Oh YJ, Zhang J, Fang ML, Fukuoka Y (2021). A systematic review of artificial intelligence chatbots for promoting physical activity, healthy diet, and weight loss. Int J Behav Nutr Phys Act.

[90] Feng J, Phillips RV, Malenica I (2022). Clinical artificial intelligence quality improvement: towards continual monitoring and updating of AI algorithms in healthcare. NPJ Digit Med.

[91] Alsareii SA, Raza M, Alamri AM (2022). Machine learning and internet of things enabled monitoring of post-surgery patients: a pilot study. Sensors (Basel).

[92] Cruz-Gonzalez P, He AWJ, Lam EP (2025). Artificial intelligence in mental health care: a systematic review of diagnosis, monitoring, and intervention applications. Psychol Med.

[93] Li YH, Li YL, Wei MY, Li GY (2024). Innovation and challenges of artificial intelligence technology in personalized healthcare. Sci Rep.

[94] Bhuyan SS, Sateesh V, Mukul N (2025). Generative artificial intelligence use in healthcare: opportunities for clinical excellence and administrative efficiency. J Med Syst.

[95] Wachter RM, Brynjolfsson E (2024). Will generative artificial intelligence deliver on Its promise in health care?. JAMA.

[96] Kilanko V (2023). The transformative potential of artificial intelligence in medical billing: a global perspective. Int J Sci Adv.

[97] O’Malley GR, Sarwar SA, Cassimatis ND (2024). Can publicly available artificial intelligence successfully identify current procedural terminology codes for common procedures in neurosurgery?. World Neurosurg.

[98] Dai HJ, Wang CK, Chen CC (2024). Evaluating a natural language processing-driven, AI-assisted international classification of diseases, 10th revision, clinical modification, coding system for diagnosis related groups in a real hospital environment: algorithm development and validation study. J Med Internet Res.

[99] Eloundou T, Manning S, Mishkin P, Rock D (2024). GPTs are GPTs: labor market impact potential of LLMs. Science.

[100] El Moheb M, Putman K, Sears O (2025). An open-architecture AI model for CPT coding in breast surgery: development, validation, and prospective testing. Ann Surg.

[101] Sulis E, Mariani S, Montagna S (2023). A survey on agents applications in healthcare: opportunities, challenges and trends. Comput Methods Programs Biomed.

[102] Gao S, Fang A, Huang Y (2024). Empowering biomedical discovery with AI agents. Cell.

[103] Nathani D, Madaan L, Roberts N (2025). MLGym: a new framework and benchmark for advancing AI research agents. arXiv.

[104] Rao R, Liu J, Verkuil R (2021). MSA Transformer. bioRxiv.

[105] Yu MK, Kramer M, Dutkowski J (2016). Translation of genotype to phenotype by a hierarchy of cell subsystems. Cell Syst.

[106] Singh D, Febbo PG, Ross K (2002). Gene expression correlates of clinical prostate cancer behavior. Cancer Cell.

[107] Fahim YA, Hasani IW, Kabba S, Ragab WM (2025). Artificial intelligence in healthcare and medicine: clinical applications, therapeutic advances, and future perspectives. Eur J Med Res.

[108] Garcia-Gomez JM, Blanes-Selva V, Alvarez Romero C (2025). Mitigating patient harm risks: a proposal of requirements for AI in healthcare. Artif Intell Med.

[109] Esmaeilzadeh P (2024). Challenges and strategies for wide-scale artificial intelligence (AI) deployment in healthcare practices: a perspective for healthcare organizations. Artif Intell Med.

[110] Kelly CJ, Karthikesalingam A, Suleyman M, Corrado G, King D (2019). Key challenges for delivering clinical impact with artificial intelligence. BMC Med.

[111] Siala H, Wang Y (2022). SHIFTing artificial intelligence to be responsible in healthcare: a systematic review. Soc Sci Med.

[112] Matheny ME, Goldsack JC, Saria S (2025). Artificial intelligence in health and health care: priorities for action. Health Aff (Millwood).

[113] Curcin V, Delaney B, Alkhatib A (2026). Learning health systems provide a glide path to safe landing for AI in health. Artif Intell Med.

[114] Jain SS, Elias P, Poterucha T (2024). Artificial intelligence in cardiovascular care-part 2: applications: JACC review topic of the week. J Am Coll Cardiol.

